# Insight into the mitochondrial unfolded protein response and cancer: opportunities and challenges

**DOI:** 10.1186/s13578-022-00747-0

**Published:** 2022-02-18

**Authors:** Ge Wang, Yumei Fan, Pengxiu Cao, Ke Tan

**Affiliations:** 1 Key Laboratory of Molecular and Cellular Biology of Ministry of Education, Key Laboratory of Animal Physiology, Biochemistry and Molecular Biology of Hebei Province, College of Life Sciences, Hebei Normal University, Hebei, 050024 China; 2 Department of Human Anatomy, Histology and Embryology, Key Laboratory of Carcinogenesis and Translational Research (Ministry of Education), State Key Laboratory of Natural and Biomimetic Drugs, Peking University Health Science Center, Beijing, 100191 China

**Keywords:** Mitochondrial unfolded protein response, Cancer, Proteostasis, Mitochondrial heat shock protein, Mitochondrial protease

## Abstract

The mitochondrial unfolded protein response (UPR^mt^) is an evolutionarily conserved protective transcriptional response that maintains mitochondrial proteostasis by inducing the expression of mitochondrial chaperones and proteases in response to various stresses. The UPR^mt^-mediated transcriptional program requires the participation of various upstream signaling pathways and molecules. The factors regulating the UPR^mt^ in *Caenorhabditis elegans* (*C. elegans*) and mammals are both similar and different. Cancer cells, as malignant cells with uncontrolled proliferation, are exposed to various challenges from endogenous and exogenous stresses. Therefore, in cancer cells, the UPR^mt^ is hijacked and exploited for the repair of mitochondria and the promotion of tumor growth, invasion and metastasis. In this review, we systematically introduce the inducers of UPR^mt^, the biological processes in which UPR^mt^ participates, the mechanisms regulating the UPR^mt^ in *C. elegans* and mammals, cross-tissue signal transduction of the UPR^mt^ and the roles of the UPR^mt^ in promoting cancer initiation and progression. Disrupting proteostasis in cancer cells by targeting UPR^mt^ constitutes a novel anticancer therapeutic strategy.

## Background

Mitochondria are the power houses of cells, but their functions extend far beyond energy metabolism. Mitochondria play a vital role in the production of metabolic intermediates, calcium homeostasis, immune responses, cell differentiation, cell death, and the maintenance of proteostasis [1, 2]. Mitochondrial function requires the maintenance of mitochondrial homeostasis, which can be disrupted by many endogenous and exogenous stimuli. Therefore, a cytoprotective stress mechanism called the mitochondrial unfolded protein response (UPR^mt^) is activated upon mitochondrial damage and promotes mitochondrial recovery. UPR^mt^ induces a transcriptional program involving numerous genes through retrograde mitochondrial-to-nucleus communication [3–5]. The UPR^mt^ establishes a stable mitochondrial environment through the refolding of unfolded and misfolded proteins, degradation of damaged proteins through mitochondrial autophagy (mitophagy), regulation of mitochondrial biogenesis, and amelioration of oxidative stress [6–8].

The UPR^mt^, as an evolutionarily conserved mitochondrial stress response mechanism, can be induced in most organisms, such as *Saccharomyces cerevisiae* (*S. cerevisiae*), *Caenorhabditis elegans* (*C. elegans*), *Drosophila*, *Homo sapiens*, and *Arabidopsis thaliana*, to maintain mitochondrial proteostasis and function [9, 10]. Although different species may have unique signaling molecules upstream of UPR^mt^, the functional categories of UPR^mt^ target genes show substantial overlap [10]. In summary, the UPR^mt^, a program for maintaining mitochondrial proteostasis, is indispensable in most organisms.

## Inducers of the UPR^mt^

### Inducers of the UPR^mt^ in *C. elegans*

Numerous studies have shown that a large number of molecules or agents can activate the UPR^mt^ (Table 1). Polyglutamine repeat protein (polyQ), an aggregation-prone protein, binds to mitochondria and triggers the UPR^mt^ [11, 12]. Exposure of nematodes to nanoplastic particles induced the UPR^mt^ in the intestine [13]. In addition, combined treatment with nanopolystyrene further enhanced the toxicity of microgravity stress to nematodes and induced UPR^mt^ [14]. Statins, cholesterol-lowering drugs targeting the mevalonate pathway, have been shown to trigger the UPR^mt^ [15]. Furthermore, a salicylic acid derivative, C8-SA, activates the UPR^mt^, and this response is associated with increased lifespan in *C. elegans* (Table 1) [16]. Through a screen of the *C. elegans* genome, a previous study showed that knockout of many mitochondrial process-related genes induced UPR^mt^, which explained the role of these genes in the maintenance of mitochondrial homeostasis (Table 2) [17]. These genes included those encoding mitochondrial large and small ribosomal subunits, transcription- and translation-related factors, tricarboxylic acid (TCA) cycle- and lipid metabolism-related proteins, mitochondrial chaperones and proteases, electron transport chain (ETC) components and ETC assembly factors (Table 2) [17–21]. The lack of respiratory chain components and assembly factors causes dysregulation of oxidative phosphorylation (OXPHOS), leading to accumulation of reactive oxygen species (ROS) and a decrease in the mitochondrial membrane potential. The decrease in the mitochondrial membrane potential acts as a signal to activate the UPR^mt^ [17].

**Table 1:** Inducers of the UPR^mt^

Inducers	Functional role	Impact	References
Abnormal respiratory chain	Dysregulation of OXPHOS	ROS accumulation, decrease in the mitochondrial membrane potential	[17]
C8-SA	Activation of DAF-16	Antioxidant stress	[16]
FUS proteinopathies	Interference of ATP synthase complex formation	Inhibition of mitochondrial ATP synthesis	[26]
Heat shock	Activation of HSF1, upregulation of mitochondrial chaperones	Maintenance of mitochondrial proteostasis	[64, 65]
Inflammation	Involvement of TNF-α	ROS accumulation, overload of damaged proteins	[24]
Microgravity stress	Not mentioned	ROS accumulation, decrease in locomotion behavior	[14]
Mitochondrial-related genes knockout	Disorder of mitochondrial function	Imbalance of mitochondrial homeostasis	[17–21]
Nanopolystyrene	Activation of ELT-2 signaling, Wnt signaling, and insulin signaling	ROS accumulation, decrease in locomotion behavior	[13]
Nicotinamide riboside	Activation of SIRT3	Antioxidant stress	[22, 23, 58]
PolyQ	Direct interaction with the outer membrane of the mitochondria	ROS accumulation, decrease in the mitochondrial membrane potential	[11, 12]
Statins	Inhibition of HMG-CoA reductase in the mevalonate pathway	Interference of mitochondrial electron carriers	[15]
TDP-43 proteinopathies	Inhibition of ATP synthesis	abnormal cristae and a loss of cristae, ROS accumulation	[27]

**Table 2:** List of mitochondrial process-related genes associated with UPR^mt^

Group	Gene name	Gene description	References
Mitochondrial large ribosomal subunits	*mrpl-1*	Mitochondrial ribosomal protein L1	[17, 20, 21]
	*mrpl-2*	Ribosomal L2 C domain-containing protein	[17, 20, 21]
	*mrpl-9*	39S ribosomal protein L9, mitochondrial	[17]
	*mrpl-11*	Putative 39S ribosomal protein L11, mitochondrial	[17]
	*mrpl-50*	39S ribosomal protein L50, mitochondrial	[17, 21]
Mitochondrial small ribosomal subunits	*mrps-2*	Mitochondrial ribosomal protein, S2	[17, 20, 21]
	*mrps-5*	Putative 28S ribosomal protein S5, mitochondrial	[17, 20, 21]
	*mrps-18b*	Mitochondrial ribosomal protein, S18b	[17, 20]
	*mrps-24*	28S ribosomal protein S24, mitochondrial	[17]
	*mrps-35*	MRP-S28 domain-containing protein	[17, 20]
Complex I ETC	*nuo-3*	NADH ubiquinone oxidoreductase	[17, 21]
	*gas-1*	Putative NADH dehydrogenase [ubiquinone] iron-sulfur protein 2	[17, 20]
	*lpd-5*	NADH dehydrogenase [ubiquinone] iron-sulfur protein 4, mitochondrial	[17, 21]
Complex V ETC	*atp-2*	ATP synthase subunit beta, mitochondrial	[17, 20, 21]
	*hpo-18*	Mitochondrial F1F0-ATP synthase, subunit epsilon/ATP15	[17, 20]
	*asb-1*	ATP synthase subunit b	[17]
Mitochondrial ETC assembly factors	*sco-1*	Putative cytochrome C oxidase assembly protein	[17, 20]
	*cox-18*	Cytochrome oxidase assembly protein	[17]
Mitochondrial import	*dnj-21*	Mitochondrial import inner membrane translocase subunit TIM14	[17, 20, 21]
	*gop-3*	SAM50-like protein gop-3	[17]
Lipid metabolism	*acdh-13*	Acyl CoA dehydrogenase	[17]
	*mecr-1*	Enoyl-[acyl-carrier-protein] reductase	[17, 21]
Translation elongation factors	*tufm-1*	Elongation factor Tu, mitochondrial	[17, 20]
	*gfm-1*	Elongation factor G, mitochondrial	[17]
Mitochondrial proteases	*spg-7*	AFG3-like protein spg-7	[17, 21]
	*atad-3*	ATPase family AAA domain-containing protein 3	[17]
Mitochondrial chaperones	*hsp-60*	Chaperonin homolog Hsp-60, mitochondrial	[17, 20, 21]
	*phb-2*	Mitochondrial prohibitin complex protein 2	[17, 20, 21]
Mitochondrial transcription	*hoe-1*	Ribonuclease Z	[17]

### Inducers of the UPR^mt^ in mammals and other animals

Mitochondrial proteostasis impairment is one of the most common inducers of the UPR^mt^ (Table 1). Inhibition of mitochondrial chaperones, proteases or electron transfer complexes strongly triggers initiation of the UPR^mt^. In addition, nicotinamide riboside, a precursor of NAD^+^, can activate UPR^mt^, accompanied by a significant increase in the level of UPR^mt^-related proteins [22, 23]. In airway smooth muscle, inflammation-induced accumulation of ROS and overload of damaged proteins activate the UPR^mt^, and the proinflammatory cytokine TNF-α plays a crucial role in this event [24]. Moreover, caloric restriction can activate the UPR^mt^ to improve mitochondrial function, and miRNAs are involved as key mediators [25]. Heat shock elicits the production of various molecular chaperones, including mitochondrial heat shock proteins (HSPs) (Table 1). As a master inducer of a class of neurodegenerative diseases, fused in sarcoma (FUS) proteinopathies, FUS interacts with the catalytic subunit of mitochondrial ATP synthase, which interferes with the formation of ATP synthase complexes and hinders the production of energy, thus triggering UPR^mt^ activation in *Drosophila* (Table 1) [26]. Additionally, mutations or dysregulation of TDP-43 can cause TDP-43 proteinopathies. Mitochondrial impairment is one of the characteristics of TDP-43 proteinopathies; morphologically, mitochondrial cristae exhibit reduced numbers and abnormal phenotypes in animal experiments and patient brain samples. In mammalian cell and *Drosophila* models, increased TDP-43 expression inhibits ATP synthesis and accelerates ROS production, thus activating the UPR^mt^ (Table 1) [27].

## Regulation of the UPR^mt^

### Mitochondrial retrograde signaling pathways in *Saccharomyces cerevisiae*

Retrograde signaling in *S. cerevisiae* is the first retrograde pathway and has been elucidated in great detail [28, 29]. The most important event in retrograde signaling in *S. cerevisiae* is the nuclear translocation of retrograde transcription factors from the cytoplasm, including Rtg1p, Rtg2p, and Rtg3p [28–30]. Rtg1p and Rtg3p are basic helix-loop-helix/leucine zipper proteins and form a heterodimer to bind to the DNA binding site R box (GTCAC), thus regulating the expression of various genes that encode mitochondrial proteins. The translocation of this heterodimeric transcription factor is partially regulated by the dephosphorylation of Rtg3p, which is mediated by Mks1p when it binds to the 14-3-3 protein Bmh1p or Bmh2p [28–30]. Rtg2p is also an activator of this retrograde signaling pathway. Rtg2p facilitates the dephosphorylation of Rtg3p by binding to Mks1p and inhibiting Mks1p to form a complex with Bmh1p/Bmh2p, a complex maintaining Rtg3p in a hyperphosphorylated state [31, 32]. Interestingly, there are currently no reports strictly related to UPR^mt^ in yeast. Although there is no signaling pathway strictly classified as UPR^mt^ in yeast, very similar mechanisms exist that repair mitochondrial dysfunction by activating the expression of nuclear genes.

### The UPR^mt^ in *C. elegans*

**Fig. 1:**
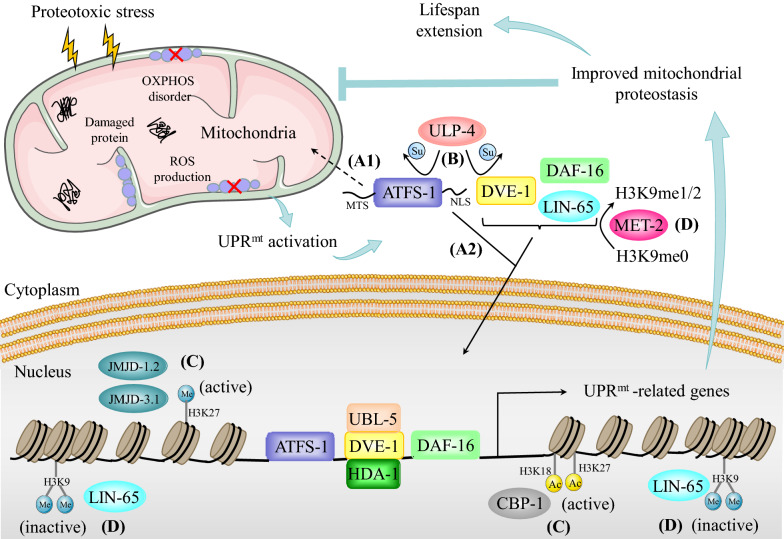
Mechanism of UPR^mt^ in *C. elegans.* When mitochondrial proteostasis is disturbed, signaling molecules of UPR^mt^ mediate mitochondria-to-nucleus retrograde communication. Transcription factors, including ATFS-1, DVE-1, and DAF-16, are activated by upstream signals and bind to the promoters of target genes, thus inducing the transcription of UPR^mt^-related genes. **A1** Under nonstress conditions, ATFS-1 enters the mitochondria through the MTS and is subsequently degraded. **A2** Under mitochondrial stress conditions, ATFS-1 is transported to the nucleus in an NLS-dependent manner. **B** ULP-4-mediated deSUMOylation of ATFS-1 and DVE-1 enhances ATFS-1- and DVE-1-dependent transcription programs. **C** JMJD-1.2-, JMJD-3.1- and CBP-1-dependent epigenetic modifications facilitate the formation of a nucleosome conformation that is conducive to transcription. **D** Additionally, MET-2- and LIN-65-mediated chromatin silencing of non-UPR^mt^ gene regions is crucial for triggering UPR^mt^. Consequently, the activation of UPR^mt^ promotes the recovery of mitochondria from damage, enhances mitochondrial function, and prolongs the lifespan of *C. elegans*

A multicellular eukaryote, *C. elegans* has been widely used as a model organism in genetics, developmental biology, neurobiology, and molecular biology. Studies using *C. elegans* as a model organism have gradually deepened our understanding of the UPR^mt^ and have revealed its components and regulatory pathways. The most well-known regulator of the UPR^mt^ in *C. elegans* is activating transcription factor associated with stress-1 (ATFS-1) (Fig. 1). The amino terminus of ATFS-1 contains the mitochondrial targeting sequence (MTS). Under normal conditions, ATFS-1 enters the mitochondrial matrix in an MTS-dependent manner through channel proteins located on the mitochondrial membrane and is degraded by the protease Lon peptidase 1 (LONP1) [4, 33]. Under mitochondrial stress conditions, a decrease in the mitochondrial transport efficiency of ATFS-1 causes it to be retained in the cytoplasm. The nuclear localization sequence (NLS) located at the carboxyl terminus of ATFS-1 mediates its nuclear transport, and after nuclear translocation, ATFS-1 functions as a transcription factor to drive the UPR^mt^ transcriptional program (Fig. 1) [17, 33]. When the ETC is disrupted, a large proportion of ATFS-1 eventually enters the nucleus. Subsequently, ATFS-1 fine-tunes the expression of OXPHOS-related genes (such as *atp-3*, *nuo-4* and *nduf-6*) and TCA cycle-related genes (such as *aco-2*, *idh-1* and *pyc-1*) in both the nucleus and mitochondria to adapt the metabolic capacity to the limited protein folding capacity, thereby reducing the load of misfolded proteins and promoting the recovery of OXPHOS [4]. In addition, when proteostasis is destroyed, DVE-1 and UBL-5 form a complex; DVE-1 then binds to the promoters of genes encoding mitochondrial chaperones, including heat shock protein 60 (HSP60), activating their transcription to maintain proteostasis (Fig. 1) [34]. Moreover, a genome-wide RNAi screen identified ULP-4 as a regulator of UPR^mt^ [35]. Knockdown of ULP-4 greatly impaired the activation of UPR^mt^, but not ER stress or the heat shock response (HSR), in *C. elegans.* Yeast two-hybrid screening identified two key substrates of ULP-4: DVE-1 and ATFS-1 [35]. Surprisingly, SUMOylation influences DVE-1 and ATFS-1 through two different molecular mechanisms. Normally, DVE-1 is SUMOylated on the K327 residue. Knockdown of ULP-4 significantly increased the SUMOylation level of DVE-1, thus impeding the nuclear translocation of DVE-1 and suppressing the activation of UPR^mt^ during mitochondrial stress [35]. ATFS-1 is another substrate of ULP-4. ULP-4 interacted with and deSUMOylated ATFS-1 at residue K326 following mitochondrial stress. Although knockdown of ULP-4 did not affect the nuclear translocation of ATFS-1, the protein stability and transcriptional activity of ATFS-1 were greatly decreased (Fig. 1) [35]. These posttranslational modifications enhance innate immunity and prolong the lifespan of nematodes. The NAD^+^-dependent protein deacetylase *sir-2.1* leads to activation of the FOXO transcription factor DAF-16 and promotes DAF-16 to induce the expression of antioxidant genes when proteostasis is dysregulated. Activation of UPR^mt^ prolongs the lifespan of nematodes after exogenous addition of the NAD^+^ precursor increases the NAD^+^ level [36].

Changes in chromatin structure also play a substantial role in the UPR^mt^ via a mechanism related to epigenetic modification [37]. H3K9 dimethylation (H3K9me2) mediated by the histone methyltransferase MET-2 and its cofactor LIN-65 silences the expression of some genes, while ATFS-1 and DVE-1 synergistically promote the transcription of UPR^mt^ genes in nonsilenced regions (Fig. 1). Interestingly, the nuclear enrichment of DVE-1 and LIN-65 is interdependent [38]. The nematode HDA-1, a homologous protein of mammalian HDAC, interacts with DVE-1 to activate the UPR^mt^ [39]. The histone demethylases JMJD-1.2 and JMJD-3.1 cooperate to promote the epigenetic transition from H3K27me3 to H3K27me1, inducing the transcription of UPR^mt^ effectors and delaying senescence in nematodes (Fig. 1) [40]. A recent study indicated that the acetyltransferase CBP-1 mediates the acetylation of histone H3 at K18 and K27, which facilitates the binding of transcription factors to the promoters of UPR^mt^ genes (Fig. 1) [41]. Although great progress has been made in understanding the regulation of UPR^mt^ in nematodes, the more precise regulatory mechanism is still unclear. The scientific question of how ATFS-1, DVE-1 and DAF-16 coordinate their functions to drive transcription programs needs to be further explored.

### The cell-nonautonomous UPR^mt^

The nervous system plays a vital role in the maintenance of organism homeostasis in *C. elegans* [42]. Previous studies have revealed that mitochondrial stress in nematode neurons can be transmitted to distal tissues and activate the UPR^mt^ in a nonautonomous manner [18, 43]. The substances released from neurons, collectively known as mitokines, are involved in activating the UPR^mt^ in distal tissue [44, 45]. Downregulation of *cco-1* in *C. elegans* neurons triggers the secretion of Wnt, subsequently leading to activation of the Wnt signaling-dependent UPR^mt^ in peripheral tissues, which regulates the organism’s mitochondrial homeostasis [18]. In *C. elegans*, neurons with abnormal respiratory chain function and ROS accumulation release serotonin, which requires UNC-31-mediated neurosecretion. Immediately after its release, serotonin acts on the distal intestine, activates the UPR^mt^ and triggers adaptive metabolic changes in response to proteotoxic stress [11]. Activation of FSHR-1 in neurons promotes the function of SPHK-1 in the intestine and consequently triggers UPR^mt^ activation in response to stress [46]. Under stimulation by neuroendocrine signals, SPHK-1 localizes to the mitochondrial membrane and catalyzes the conversion of SPH to S1P, which participates in the activation of the cell-nonautonomous UPR^mt^ as an early event. SPHK-1 mutants lacking kinase activity or mitochondrial localization ability cannot effectively induce UPR^mt^ [47]. In addition, neural circuits mediated by three types of sensory neurons (ASK, AWA and AWC) and an interneuron (AIA) are involved in the sensing and communication of neuronal mitochondrial dysfunction, in which the neuropeptide FLP-2 plays a crucial role [43]. In summary, the nervous system of *C. elegans* systematically coordinates the cellular nonautonomous UPR^mt^ through the release of endocrine signals called mitokines, which facilitate the communication of information across cells and tissues to regulate metabolism, maintain homeostasis and prolong lifespan. Moreover, communication between germline and intestinal cells can also be conducted through endocrine signals, and translational repression of CYC-2.1 mediated by the RNA-binding protein GLD-1 in germline cells is involved in this process [48].

### The UPR^mt^ in *Drosophila*

Research on UPR^mt^ with *Drosophila* as the experimental material has made a significant contribution to our current understanding of UPR^mt^. A previous study showed that mild muscle mitochondrial damage maintains mitochondrial function, inhibits the deterioration of muscle structure and function, and extends the lifespan of *Drosophila* [49]. The mechanism underlying this phenomenon involves redox-dependent induction of UPR^mt^-related genes and systemic repression of insulin signaling via the *Drosophila* ortholog of insulin-like growth factor-binding protein 7 (IGFBP7) [49]. Phosphoglycerate mutase 5 (PGAM5), a mitochondrial phosphatase, is cleaved by the rhomboid protease PARL and released from membranes during mitochondrial stress. Numerous studies have indicated that PGAM5 is involved in the regulation of mitochondrial homeostasis not only by activating mitochondrial biogenesis and mitophagy but also by inducing excessive mitochondrial fission and different types of cell death, such as apoptosis and necroptosis [50–52]. In *Drosophila*, the mitochondrial membrane protein PGAM5 senses mitochondrial stress and activates the transcription factor FoxO through ASK1 and JNK [53]. Persistent activation of FoxO upregulates the expression of multiple chaperones, thereby promoting the recovery of mitochondria and extending the lifespan of organisms [53].

### The UPR^mt^ in mammals

**Fig. 2:**
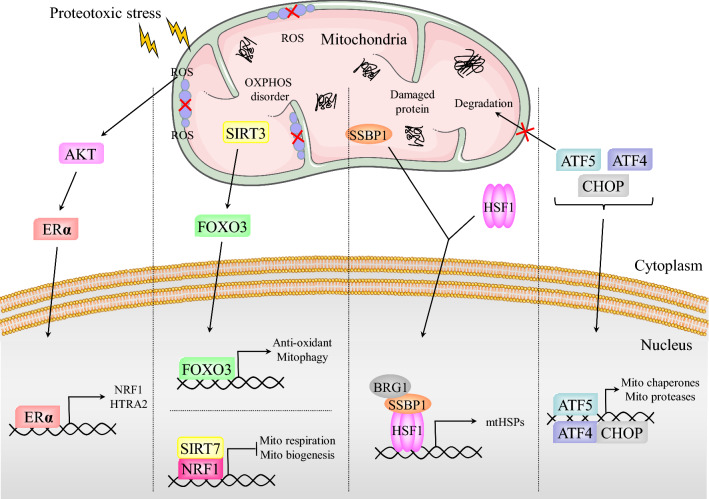
Mechanism of UPR^mt^ in mammals. Upon ROS accumulation in IMS, the AKT-ERα axis is activated to trigger the transcription of NRF1 and HTRA2 in response to IMS damage. When ATF5 senses mitochondrial disorders, it translocates to the nucleus together with ATF4 and CHOP to synergistically promote the expression of mitochondrial chaperones and proteases. HSF1 forms a complex with SSBP1 and recruits the chromatin remodeling factor BRG1, consequently inducing the expression of mtHSPs. The SIRT3-FOXO3 axis is involved in antioxidant stress and mitophagy. The interaction of NRF1 and SIRT7 inhibits mitochondrial respiration and biogenesis, thereby reducing the load of damaged proteins. In summary, the mammalian UPR^mt^ promotes mitochondrial recovery and maintains proteostasis through a variety of pathways

The factors and mechanisms that regulate UPR^mt^ in mammals are more complex than those in *C. elegans*. However, our understanding of the mammalian UPR^mt^ is far less advanced than our understanding of the *C. elegans* UPR^mt^. Proteostasis is essential for cell survival, and the ClpXP complex protease plays a key role in maintaining proteostasis. The ClpXP complex is composed of two subunits: ClpX and the caseinolytic mitochondrial matrix peptidase proteolytic subunit (ClpP). Overexpression of ClpX leads to upregulation of some genes associated with UPR^mt^, suggesting that ClpX is involved in the initiation of UPR^mt^ [54]. The mitochondrial matrix peptidase ClpP not only maintains mitochondrial proteostasis by degrading unfolded and misfolded proteins but also mediates UPR^mt^ induction. A decrease in the ClpP level weakens the UPR^mt^ in mouse cells, resulting in mitochondrial dysregulation [55]. The results of previous studies indicate that CHOP is activated by UPR^mt^. In turn, CHOP transcriptionally upregulates the molecular chaperones HSP10 and HSP60 to increase the protein folding ability of mitochondria (Fig. 2) [56]. Notably, CHOP is also involved in regulating the endoplasmic reticulum unfolded protein response (UPR^ER^). However, disruption of mitochondrial proteostasis does not induce the UPR^ER^, indicating that CHOP triggers the expression of only mitochondria-localized stress proteins. The selective and specific induction of CHOP during the UPR^mt^ may be caused by the activation of AP-1 [56]. Furthermore, ATF4 is reported to be involved in the UPR^mt^ by resetting cellular metabolism [5]. More importantly, as a mammalian homolog of ATFS-1, activating transcription factor 5 (ATF5) is similar to ATFS-1 in its regulatory mechanisms and transcriptional programs induced in the UPR^mt^ process (Fig. 2) [3]. ATF5 also possesses an MTS and an NLS. ATF5 is transported to mitochondria via the TIM-TOM complex under nonstress conditions by recognition of its MTS and is subsequently degraded. Under overload of misfolded and unfolded proteins as well as protein aggregation in mitochondria, ATF5 translocates to the nucleus by recognition of its NLS and upregulates a variety of molecular chaperones and proteases to promote mitochondrial recovery [3].

Previous studies revealed that the deacetylase SIRT3 protects cells from mitochondrial damage through upregulation of antioxidant activity and mitophagy (Fig. 2) [57]. Mechanistically, SIRT3 deacetylates FOXO3 at K271 and K290 in response to the accumulation of ROS and a decrease in the mitochondrial membrane potential, and the resulting activated FOXO3 is then redistributed into the nucleus and transcriptionally upregulates antioxidant- and mitophagy-related genes (Fig. 2). Among these events, FOXO3-mediated upregulation of PGC-1α and SOD2 plays a critical role [58, 59]. As an important mediator of antioxidant stress in the UPR^mt^, SOD2 plays an indispensable role in reducing the level of mitochondrial ROS [60]. Additionally, activation of the SIRT1/UPR^mt^/SOD signaling axis is involved in the elimination of excess mitochondrial ROS [36]. In breast cancer cells, the accumulation of misfolding-prone proteins in the mitochondrial intermembrane space (IMS) interferes with ETC homeostasis, leading to an increase in the level of ROS in the IMS and subsequently triggering the activation of estrogen receptor α (ERα or ESR1) (Fig. 2). ERα regulates the expression of its target genes NRF1 and HTRA2. The protease HTRA2 degrades misfolded proteins in the IMS to ameliorate IMS stress [61]. Upon protein folding stress, NRF1, a major mitochondrial regulator, binds to the histone deacetylase SIRT7 (Fig. 2). NRF1 transports SIRT7 to the promoter region of target genes and suppresses their transcription by compressing the local region of chromatin [62]. The NRF1-SIRT7 complex regulates energy metabolism by inhibiting mitochondrial respiration and balances suboptimal protein folding and degradation capabilities by inhibiting mitochondrial biogenesis (Fig. 2) [62]. Inhibition of protein translation is an important component of the UPR^mt^. In response to the disruption of proteostasis, cells rapidly express high levels of mitochondrial chaperones, while UPR^mt^ reduces the biogenesis of mitochondria-localized proteins by impeding pre-RNA processing and translation. Through this mechanism, a balance between the enhanced protein folding capacity and the reduced protein folding load is achieved, enabling accelerated repair of cellular injury [63].

The mammalian histone demethylases PHF8 and JMJD3, homologs of *C. elegans* JMJD-1.2 and JMJD-3.1, respectively, induce the expression of UPR^mt^-related genes by altering epigenetic patterns [40]. Mammalian CBP/p300-dependent H3K18Ac and H3K27Ac participate in the formation of an active chromatin state, thereby promoting the transcription of UPR^mt^ genes [41]. In addition, inhibition of HDAC1/2 suppresses the transcriptional program mediated by UPR^mt^ in mammalian cells [39].

Our previous studies have shown that heat shock factor 1 (HSF1), a master regulator of the heat shock response (HSR), is also involved in UPR^mt^ [64]. Activated HSF1 recruits mitochondrial single-stranded DNA binding protein 1 (SSBP1) to the promoters of cytoplasmic and mitochondrial chaperone genes (Fig. 2). Subsequently, the HSF1-SSBP1 complex recruits the chromatin remodeling factor BRG1, thereby promoting the formation of the chromatin remodeling complex [64]. The upregulation of the mitochondrial chaperones HSP10, HSP60 and mitochondrial heat shock protein 70 (mtHSP70) induced by HSF1 participates in UPR^mt^ to combat proteotoxicity stress (Fig. 2) [64–66]. RNA-seq analysis identified that interferon-α inducible protein 6 (IFI6) confers radioprotection in skin cells. IFI6 translocates into nucleus in response to radiation and interacts with SSBP1 to increase the transcriptional activity of HSF1 [67]. Recently, a mitochondrial stress-specific variant of HSF1, dephosphorylated by the PP2A complex, was found in *C. elegans* upon ETC impairment and exhibited a protective role in age-related dysfunction of proteostasis by selectively upregulating the expression of small HSPs [68]. Therefore, it will be interesting to explore whether mitochondrial localization of HSF1 exists in mammalian cells. Overall, although increased research has gradually improved the framework of UPR^mt^ regulation in mammals, the details of the regulation of each branch of UPR^mt^ are still unclear. With the increasing depth of research, more proteins involved in UPR^mt^ will be discovered, and the potential relationships among branches will be clarified.

## Relationships between Ca^2+^ regulation, mitophagy, ISR and UPR^mt^

### The UPR^mt^ and Ca^2+^ regulation

Calcium, as a key intracellular second messenger, plays a pivotal role in many physiological and pathological processes. Ca^2+^ transfer from the endoplasmic reticulum (ER) to mitochondria is the main source of mitochondrial Ca^2+^, which is mediated by mitochondria-associated membranes (MAMs). Ca^2+^ regulation in mitochondria is involved in various biological processes, including ATP synthesis and metabolism [69, 70]. Previous studies have shown that the decline in brain metabolic activity during aging is associated with mitochondrial dysfunction. Disorders of OXPHOS, imbalanced Ca^2+^ buffering, and dysregulation of UPR^mt^-related proteins lead to neuronal decline during aging [71]. Under ER stress, Ca^2+^ is transported to mitochondria through the MAM, which ultimately leads to increased expression of the mitochondrial protease LONP1 [72, 73]. Recent studies have demonstrated that LONP1 is required for the maintenance of mitochondrial proteostasis and gene expression. LONP1 depletion leads to loss of mitochondrial DNA (mtDNA), impaired ribosome biogenesis, accumulation of insoluble protein aggregates in the matrix, stabilization of PINK1, and activation of the integrated stress response (ISR) [74]. However, the exact relationship between mitochondrial Ca^2+^ signaling and UPR^mt^ has not been elucidated. The potential crosstalk between Ca^2+^ regulation in mitochondria and UPR^mt^ urgently needs to be explored.

### The UPR^mt^ and mitophagy

Mitochondria are the first-line defense of cells under various stresses. Dysfunctional mitochondria can be repaired by a complex set of adaptive responses, such as mitochondrial biogenesis, mitochondrial fission/fusion, UPR^mt^ and mitophagy. UPR^mt^ and mitophagy are two major axes that maintain mitochondrial proteostasis [75]. As discussed above, UPR^mt^ facilitates the recovery and survival of cells by inducing the expression of mitochondrial chaperones, proteases, antioxidant genes and protein import and assembly factors. When damaged mitochondria cannot be accurately repaired by a small-scale stress response, such as UPR^mt^, dysfunctional mitochondria can be removed by mitophagy. Mitophagy targets damaged mitochondria through autophagosome phagocytosis and lysosomal degradation to ensure that they are cleared before they become toxic to cells [76–78]. The molecular mechanisms of PINK1/Parkin- and BNIP3/NIX-regulated mitochondrial clearance have previously been reviewed in detail [76–78]. Therefore, it is speculated that UPR^mt^ appears to be activated prior to mitophagy. Inconsistent with this conclusion, a recent study revealed that UPR^mt^ was a downstream pathway of mitophagy [79, 80]. Both UPR^mt^ and mitophagy were slightly activated in LPS-treated cardiomyocytes to sustain mitochondrial function. Treatment with urolithin A, an inducer of mitophagy, significantly reduces sepsis-mediated heart injury by restoring mitochondrial function without influencing the UPR^mt^ [80]. However, deletion of FUN14 domain containing 1 (FUNDC1), a mammalian mitophagy receptor localized on the outer mitochondrial membrane, greatly increased the expression of UPR^mt^-related genes in LPS-treated mouse hearts [80]. In contrast, the enhancement of UPR^mt^ by oligomycin effectively alleviated sepsis-induced mitochondrial damage and myocardial dysfunction. This cardioprotective effect was not evident in FUNDC1 CKO mice [80]. Moreover, when UPR^mt^ was suppressed, mitophagy-mediated cardiac protection was partially attenuated [80]. In summary, UPR^mt^ and mitophagy are important quality control mechanisms. However, the precise molecular mechanism by which mitophagy modulates UPR^mt^ has not been clarified. Whether other classic mitophagy signaling pathways and regulatory proteins mediate UPR^mt^ also needs to be further verified by in vivo and in vitro experiments.

### The UPR^mt^ and integrated stress response (ISR)

The ISR is also an evolutionarily conserved cellular signaling pathway that facilitates cells and tissues to cope with various stresses, such as ER stress, heme deficiency, amino acid starvation, viral infection and hypoxia [81–83]. A large amount of evidence from mammals highlights that the ISR is the core element of UPR^mt^. In mammalian cells, ISR assists in the regulation of UPR^mt^, and its molecular mechanisms depend on the phosphorylation of the α subunit of eukaryotic translation initiation factor 2 (eIF2α), thereby suppressing the ability of eIF2 to transmit methionylated initiator transfer RNA (Met-tRNA_i_) to ribosomes [84]. The ancestral kinase general control nonderepressible 2 (GCN2) mediates phosphorylation of eIF2α during nutrient deprivation in *S. cerevisiae*. GCN-2-dependent phosphorylation of eIF2α is necessary for the development and extension of lifespan in *C. elegans*. Depletion of GCN-2 could significantly upregulate the expression of mitochondrial chaperones to activate UPR^mt^, suggesting that GCN-2-dependent translation attenuation is a parallel signaling pathway to maintain mitochondrial proteostasis during mitochondrial stress. In mammalian cells, four kinases have been identified to phosphorylate eIF2α, including GCN2, PKR-like endoplasmic reticulum kinase (PERK), protein kinase R (PKR), and heme-regulated inhibitor (HRI), in response to different forms of cellular stress [84, 85]. GCN2 is associated with ribosomes and activated by amino acid depletion. PERK, an ER membrane protein, is stimulated by the presence of unfolded or misfolded proteins in the ER. PKR is activated by the accumulation of double-stranded RNA derived from mitochondria in the cytoplasm, thus promoting eIF2α phosphorylation-mediated ISR [86]. HRI activity is regulated by the depletion of heme [84, 85, 87]. Recently, two outstanding studies revealed that mitochondrial stress induced HRI activation to promote eIF2α phosphorylation even in the presence of full heme [88, 89]. In addition, they found that OMA1, a protease localized on the inner mitochondrial membrane, cleaved the DELE1 protein. A fragment of DELE1 accumulated in the cytosol and then interacted with and phosphorylated eIF2α. Inhibition of the OMA1-DELE1-HRI signaling pathway induced the expression of specific molecular chaperones [88, 89]. These studies further established crosstalk between mitochondrial dysfunction and ISR. Phosphorylation of eIF2α leads to a reduction in the overall translation of protein synthesis to adapt to environmental changes. Paradoxically, several mRNAs containing upstream open reading frames (uORFs) can be selectively translated during ISR [81, 82]. The mRNA sequences of the transcription factors CHOP, ATF4 and ATF5 contain uORFs and require eIF2α phosphorylation to initiate their translation. These transcription factors are involved not only in the ER stress response but also in the regulation of UPR^mt^. The accumulation of unfolded proteins in the mitochondrial matrix of mammalian cells leads to CHOP-dependent transcriptional upregulation of mitochondrial chaperones and proteases but not ER stress proteins [90]. CHOP binding elements were identified in some gene promoters, such as HSP60, HSP10, mtDNAJ, ClpP, YME1L1, MPPβ, TIM17A, NDUFB2, endonuclease G and thioredoxin 2 [90, 91]. Moreover, four different mitochondrial stressors, doxycycline, actinonin, FCCP and MitoBloCK, significantly activated ATF4 to modulate ISR activation, reduce mitochondrial ribosomal proteins and suppress mitochondrial translation [5]. A recent study revealed that the noncanonical initiation factors eIF2D and DENR also participated in the translational induction of ATF4 during ISR [92, 93]. In contrast to ATF4, ATF5 seems to directly participate in the UPR^mt^ process [3, 6]. Silencing ATF5 inhibited the induction of UPR^mt^-related genes during mitochondrial stress. Interestingly, overexpression of ATF5, but not ATF4, in worms lacking ATFS-1 restored the ability to induce the expression of HSP60 [3, 6, 94]. All the above studies indicated the overlapping network between the ISR and the UPR^mt^ through the specific activation of CHOP, ATF4 and ATF5.

The imbalance of the ISR pathway is related to a variety of diseases ranging from neurodegenerative diseases to tumors, reflecting the importance of cell stress adaptation to maintain health [81–83]. The crosstalk between ISR and UPR^mt^ promotes tumor progression. GCN2-eIF2α-ATF4 axis has been shown to enhance tumor cell proliferation by maintaining metabolic homeostasis [95]. Additionally, the GCN2-eIF2α-ATF4 pathway also induces the expression of xCT, which promotes the synthesis of glutathione (GSH), which is involved in ferroptosis and ultimately leads to cisplatin resistance in gastric cancer cells [96]. However, there are still some unresolved problems [97]. For example, the consequences of ISR activation in the mitochondrial stress response need to be further explored. Although the initial purpose of ISR and the mitochondrial stress response is to protect cells against stress and avoid death, which is more important for protective effects? An in-depth understanding of the specific roles and cross-pathways of CHOP, ATF4 and ATF5 in UPR^mt^ and ISR may provide unexpected answers.

## The UPR^mt^ and cancer

The initiation and development of cancer is a multistep process that involves the acquisition of diverse functions, such as resistance to cell death, prevention of growth inhibition, and activation of proliferation signals [98]. Moreover, cancer cells are constantly under intracellular and extracellular pressure. Mutations in mtDNA, changes in metabolism, alterations in energy and oxygen requirements, and overload of mitochondrial unfolded and misfolded proteins lead to mitochondrial dysfunction. To manage various stresses, activation of the UPR^mt^ in cancer cells maintains proteostasis and regulates metabolic reactions. Furthermore, mtDNA mutations mediate cancer metastasis by activating the UPR^mt^ [99]. Notably, not all mtDNA mutations can be exploited by cancer cells, and only those specific genomic mtDNA landscapes that activate the UPR^mt^ can be utilized by metastatic cancers [99–101].

Numerous studies have shown that activation of the UPR^mt^ is indispensable for the development and progression of cancer [72, 82]. The UPR^mt^ promoted by mitohormesis in cancer cells plays a critical role in stimulating the invasion and metastasis of cancer cells [102]. For example, high expression of UPR^mt^-related genes is significantly associated with poor overall survival and metastasis-free survival in breast cancer patients [102]. Evidence supporting the upregulation of UPR^mt^ components in breast cancer suggests that UPR^mt^ activation is involved in the progression of breast cancer [103]. In addition, single-nucleotide polymorphisms in genes encoding UPR^mt^ components are associated with an increased risk of head and neck cancer [104]. Strikingly, under mitochondrial stress, UPR^mt^ mediates the secretion of the mitokine GDF15, which in turn promotes the invasion of thyroid cancer cells [105].

In principle, the UPR^mt^ facilitates cancer progression by inhibiting cancer cell death and promoting cancer growth. In the following section, we introduce the role of the signaling molecules and several proteins in the UPR^mt^ transcriptional program in cancer development. A further understanding of the signaling molecules in the UPR^mt^ and the role of UPR^mt^-related proteins in cancer development is expected to provide a new therapeutic strategy for cancer.

### The roles of upstream signaling molecules in the UPR^mt^ in cancer

Many signaling molecules in the UPR^mt^, including components of the CHOP/ATF4/ATF5, ERα and HSF1-SSBP1 axis, play an important role in tumorigenesis. These molecules promote tumorigenesis through various mechanisms in many tumors.

#### ATF5 and cancer

**Fig. 3:**
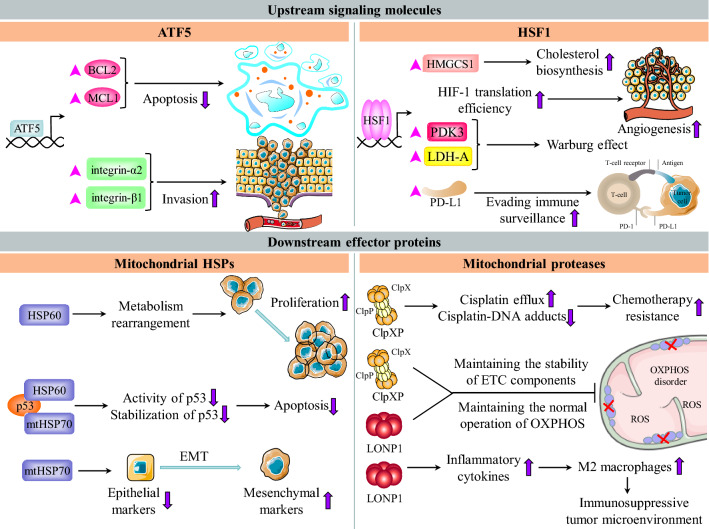
The roles of UPR^mt^ components in cancer. The upstream signaling molecules of UPR^mt^ play an important role in tumorigenesis. ATF5 transcribes BCL2 and MCL1 to inhibit cancer cell apoptosis. ATF5 upregulates integrin-α2 and integrin-β1 to promote invasion and migration. HSF1 suppresses the immune system by inducing PD-L1. HSF1 cooperates with PARP13 and PARP1 to repair the genome. The downstream effector proteins of UPR^mt^ are conducive to the progression of cancer. HSP60 promotes the proliferation of cancer cells by regulating metabolic pathways such as glycolysis and the TCA cycle. mtHSP70 is involved in epithelial mesenchymal transition. HSP60 and mtHSP70 synergistically inhibit p53 to prevent it from exerting antitumor effects, thereby promoting the survival of cancer cells. ClpXP maintains the stability of mtDNA and genomic DNA, thus reducing the sensitivity to chemotherapy. LONP1 boosts the activation and M2 polarization of macrophages, thereby creating an immunosuppressive tumor microenvironment. ClpXP and LONP1 coordinately regulate mitochondrial bioenergetics in cancer

**Fig. 4:**
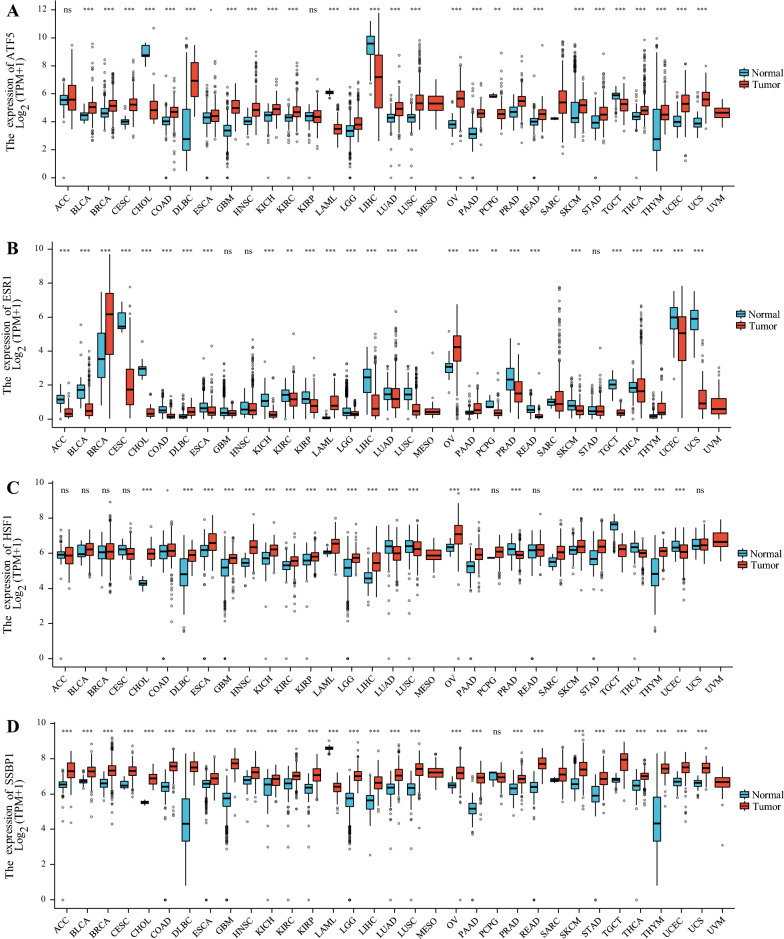

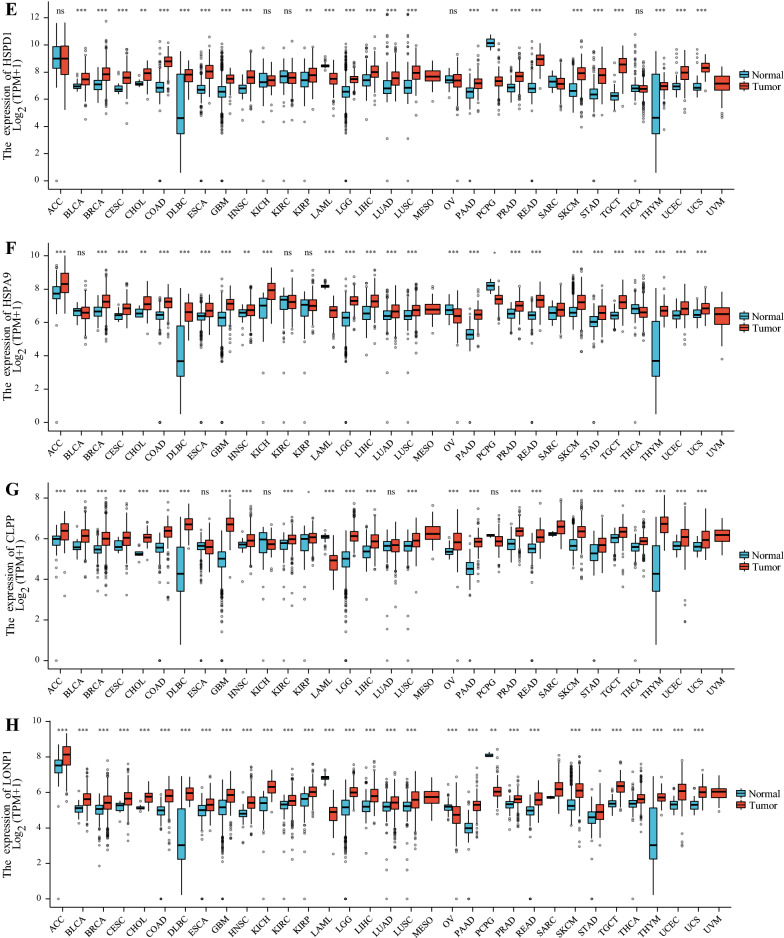
Pan-cancer analysis of the expression status of ATF5 (**A**), ERα (**B**), HSF1 (**C**), SSBP1 (**D**), HSP60 (**E**), mtHSP70 (**F**), ClpP (**G**) and LONP1 (H) in different cancers compared with adjacent normal tissues according to the TCGA and GTE_X_ databases. The ggplot2 (3.3.3) package in R software (3.6.3) was used, and TPM represents transcription per million. *P < 0.05, **P < 0.01, ***P < 0.001

Previous studies have shown that ATF5 expression is significantly upregulated in a variety of cancers, such as epithelial ovarian cancer, glioblastoma, pancreatic cancer and chronic myeloid leukemia (Figs. 3, 4A) [106–109]. One mechanism underlying the upregulation of ATF5 expression in cancer is the alteration of epigenetic modification. For example, the methylation level in the promoter region of the *ATF5* gene in glioma is significantly reduced compared with that in normal tissues. Moreover, this reduction in the methylation level is accompanied by a reduction in glioma differentiation [110]. In addition, upregulation of ATF5 transcription by the BCR-ABL/PI3K/AKT/FOXO4 signaling pathway and CREB3L2 is responsible for the high expression level of ATF5 in cancer [109, 111]. Mechanistically, ATF5 transcriptionally upregulates the expression of antiapoptotic proteins BCL2 and MCL1, hindering the apoptosis of cancer cells and reducing their chemosensitivity (Fig. 3) [106, 108, 111]. Activated ATF5 triggers the transcription of mTOR, a negative regulator of autophagy, inhibiting autophagy in cancer cells [109]. Additionally, ATF5-induced upregulation of integrin-α2 and integrin-β1 is conducive to cancer cell invasion (Fig. 3) [107]. In a previous study, a dominant-negative ATF5 mutant (DN-ATF5) lacking DNA binding ability, which blocked some signaling molecules in cells, was synthesized. The application of DN-ATF5 in cancer cell lines revealed its efficacy in reducing cancer cell viability [112]. Due to the promotive role of ATF5 in cancer, ATF5 expression is positively correlated with cancer progression in epithelial ovarian carcinomas and glioma [106, 111]. Targeting ATF5 is a potential strategy to kill cancer cells.

#### ERα and cancer

The contributions of ERα, an important component of the UPR^mt^, to tumors range far beyond its function in the UPR^mt^. As a member of the ER family, ERα has been indicated to play a critical role in a variety of tumors (Fig. 4B) [113]. ERα is expressed in 70% of breast tumors but in less than 10% of normal breast epithelium [114, 115]. One molecular mechanism underlying the increased ERα expression is that USP7 binds and deubiquitinates ERα to increase its stability [114]. Thus, mechanistically, ERα may be involved in tumorigenesis by regulating cell metabolism- and proliferation-related genes [116]. In prostate cancer, ERα facilitates the upregulation of genes related to epithelial-mesenchymal transition (EMT), thereby promoting the invasion and migration of cancer cells [117]. Due to the effect of ERα on tumor growth and invasion, ERα has been used as an effective target for endocrine cancer therapy [114, 115].

#### SSBP1, HSF1 and cancer

Previous studies have shown that the expression level of SSBP1 is upregulated in colorectal cancer (Fig. 4D) [118, 119]. Knockdown of SSBP1 leads to a decrease in mitochondrial content, suggesting that SSBP1 may promote cancer progression by influencing mitochondrial biogenesis [118]. In colorectal cancer, IL-6-STAT3-FOXP1 axis-mediated transcriptional activation of SSBP1 is beneficial for cancer cell proliferation and tumor growth [119]. Mechanistically, activated SSBP1 promotes mitochondrial biogenesis, enhances ROS production and activates the Akt/mTOR signaling pathway, resulting in telomerase activation and telomere elongation [119]. Therefore, upregulation of SSBP1 in tumor tissue predicts poor prognosis in patients with colorectal cancer [119]. Moreover, in non-small-cell lung cancer, SSBP1 enhances the resistance of cancer cells to ionizing radiation by inhibiting apoptosis [120]. CircZFR, a circular RNA, interacts with SSBP1 to promote the assembly of the CDK2/cyclin E1 complex. Subsequently, the activated CDK2/cyclin E1 complex phosphorylates Rb, thus releasing E2F1 from Rb-mediated inhibition. Consequently, E2F1 transcribes target genes to promote the G1/S transition and proliferation of cervical cancer cells [121]. However, in highly metastatic triple-negative breast cancer, SSBP1 expression is downregulated, and a low expression level of SSBP1 is associated with poor patient prognosis [122]. Low expression of SSBP1 leads to a decrease in the mtDNA copy number, thus enhancing calcineurin-dependent retrograde signaling and inducing c-Rel-mediated transcription of TGF-β. In turn, TGF-β drives EMT and metastasis of breast cancer cells [122]. The evidence indicating that SSBP1 can play either a procancer or anticancer role in different types of cancer suggests that further exploration of the function of SSBP1 in cancer development could deepen our understanding of cancer.

In addition to maintaining mitochondrial proteostasis, HSF1 can also participate in cancer initiation, development and progression by modulating the tumor microenvironment, inhibiting apoptosis, repairing the genome, promoting cell proliferation and migration and reprogramming metabolism (Figs. 3, 4C) [66, 123]. For example, PIM2-mediated activation of HSF1 induces transcriptional upregulation of PD-L1 to suppress the immune system, enabling cancer cells to evade immune surveillance (Fig. 3) [124]. In addition, HSF1 is involved in angiogenesis, which is accompanied by HuR-mediated enhancement of HIF-1 translation efficiency (Fig. 3) [125]. Consistent with its oncogenic roles, HSF1 is highly expressed in a variety of cancers (Fig. 4C). A high level of HSF1 expression predicts disease progression and a shortened survival time in patients with different types of cancer [66, 126]. Because of the dependence of cancer cells on HSF1, HSF1 can be used as an effective prognostic biomarker and is an attractive therapeutic target. Numerous screening studies have been performed to identify small-molecule inhibitors of HSF1 as next-generation anticancer chemotherapeutics. However, to date, no selective small molecule HSF1 inhibitors have been validated for clinical use. A recent study identified Direct Targeted HSF1 InhiBitor (DTHIB), a new HSF1 inhibitor that directly binds to the DNA binding domain of HSF1 and selectively promotes its degradation in the nucleus of cancer cells. More importantly, DTHIB significantly suppressed the HSF1 cancer gene signature and greatly inhibited tumor growth in mice [127]. In addition, our previous study revealed the mechanism by which cyclosporin A suppresses cancer progression by inhibiting HSF1 activity [128]. Cyclosporin A-mediated activation of ERK1/2, GSK3β and CK2 leads to phosphorylation of HSF1 at Ser303 and Ser307, which interferes with the formation of the HSF1-SSBP1 complex and reduces transcriptional activity of HSF1, resulting in the downregulation of HSP expression and inducing the death of cancer cells [128]. As HSF1 plays a pleiotropic role in cancer, its dysregulated expression in cancer and its relationship with the prognosis of cancer patients imply that HSF1 could be utilized as a biomarker for patient prognosis and a promising molecular target for cancer treatment and chemoprevention.

### The roles of downstream effector proteins in the UPR^mt^ in cancer

The transcriptional program induced by UPR^mt^ influences multiple aspects of tumorigenesis. Cancer cells need these transcripts to maintain proteostasis and mitochondrial function. Among the proteins encoded by UPR^mt^-regulated genes, mitochondrial chaperones and proteases perform an indispensable function. In this chapter, we focus on the role of UPR^mt^-induced molecular chaperones and proteases in tumors.

#### Mitochondrial HSPs and cancer

HSP60 (HSPD1) and mtHSP70 (HSPA9) are mitochondria-localized cytoprotective proteins and are the main molecular chaperones induced by UPR^mt^. Accumulating studies have shown that the occurrence and development of cancer require HSP60 and mtHSP70, which can assist the refolding of unfolded and misfolded proteins and promote the depolymerization of aggregated proteins [129]. Moreover, increasing evidence indicates that the levels of HSP60 and mtHSP70 are significantly increased in a variety of tumors (Fig. 4E, F) [129–136]. In cancer cells, one mechanism underlying HSP60 overexpression is the mediation of its transcriptional regulation by the proto-oncogenes c-MYC and HSF1 [65, 66, 132]. In ovarian cancer, NF-κB p65 binds to the promoter of mtHSP70 to transcriptionally upregulate its expression [137].

HSP60 orchestrates various cell survival programs in cancer. For example, HSP60 binds and stabilizes survivin, protecting it from degradation and thus enabling it to play an inhibitory role in apoptosis [138, 139]. The molecular chaperone complex containing HSP60 interacts with cyclophilin D, a component of the mitochondrial permeability transition pore, inhibiting cyclophilin D-dependent cancer cell death [140]. Additionally, the interaction between HSP60 and p53 reduces the stability and activity of p53 to antagonize caspase-mediated apoptosis (Fig. 3) [138]. In the cytoplasm, HSP60 interacts with IKK to boost activation-related serine phosphorylation of IKK, thus activating the prosurvival IKK/NF-κB pathway [141]. A previous study demonstrated that HSP60 modulated protein translation to facilitate the growth of ovarian cancer and glioblastoma, which requires the AMPK/mTOR pathway [142, 143]. In addition, a recent study revealed the relationship between the HSP60 expression level and tumor lymph node metastasis, and high levels of HSP60 have been linked to resistance to hormone therapy in prostate cancer [133]. Furthermore, HSP60 regulates a variety of metabolic processes, including glycolysis and the TCA cycle (Fig. 3). For example, HSP60 promotes multiple myeloma development via metabolic reprogramming [144]. HSP60 has also been shown to play a role in the extracellular environment of cancer cells. Secretion of HSP60 requires exosomes and lipid rafts [145]. In fibrosarcoma, HSP60 is transported extracellularly via the ER-Golgi secretory pathway and is therefore involved in modulating the tumor microenvironment [146].

Knockdown of mtHSP70 induces the death of melanoma cells via a mechanism related to the MEK/ERK signaling pathway and the mitochondrial permeability regulators cyclophilin D and ANT [147]. In addition, previous studies have shown that depletion of mtHSP70 stimulates the death of KRAS mutant pancreatic ductal adenocarcinoma and colon cancer cells via a mechanism associated with an increase in mitochondrial membrane permeability [148]. Under hypoxic conditions, mtHSP70 interacts with HIF-1 and colocalizes with HIF-1 to the outer mitochondrial membrane. Subsequently, VDAC1 is truncated and activated, endowing cancer cells with resistance to apoptosis induced by chemotherapy [149]. In various types of thyroid cancer, upregulation of mtHSP70 promotes the proliferation of cancer cells, while inhibition of mtHSP70 induces cell cycle arrest [150, 151]. In tumor cells with mutations in KRAS or BRAF, mtHSP70 facilitates the interaction between PP1α and MEK1/2, which modulates MEK/ERK signaling activity, thereby promoting tumor cell proliferation. In addition, mtHSP70, as a negative regulator of the Raf/MEK/ERK signaling pathway, suppresses the anticancer function of Raf/MEK/ERK signaling [152, 153]. In oral squamous cell carcinoma, mtHSP70 is secreted by cancer cells in an autocrine manner; subsequently, extracellular mtHSP70 binds to PDPN, which plays a role in cell adhesion, participating in regulating the growth and invasiveness of cancer cells [154]. Overexpression of mtHSP70 modulates the activity of the PI3K/AKT signaling pathway to accelerate tumor EMT, which is accompanied by a decrease in epithelial markers and an increase in mesenchymal markers [134]. High expression of mtHSP70 also promotes the stemness of cancer cells [155]. In hepatocellular carcinoma (HCC), overexpression of mtHSP70 is closely associated with venous infiltration and disease progression [135]. Furthermore, mtHSP70 is involved in cancer metastasis by activating hnRNP-K and inactivating p53 (Fig. 3) [156].

Taken together, these observations indicate that the mitochondrial chaperones HSP60 and mtHSP70 rely on their protein-folding ability to maintain the stability of master signaling pathways in cancer cells, thus facilitating invasion and migration. HSP60 and mtHSP70 contribute to the survival of cancer cells by inhibiting proapoptotic proteins and activating antiapoptotic proteins (Fig. 3). Thus, HSP60 and mtHSP70 are widely exploited by various cancers (Fig. 4E, F). Accumulating studies have demonstrated that high expression of HSP60 and mtHSP70 is significantly associated with poor patient outcomes in various types of cancer [157–163]. Therefore, the levels of HSP60 and mtHSP70 can be used as prognostic indicators in cancer patients. Moreover, HSP60 and mtHSP70 are employed as potential therapeutic targets due to their diagnostic role [164, 165]. For example, shRNA-mediated silencing of HSP60 inhibits the growth of HCC [166]. An anti-HSP60 antibody exhibited cytotoxicity in ovarian cancer cells. Notably, combined downregulation of HSP60 and treatment with chemotherapy exhibits a significant synergistic tumoricidal effect [167]. Previous studies have shown the efficacy of SHetA2 and PRIMA-1^MET^ in the treatment of ovarian cancer. SHetA2, a small-molecule drug, interferes with the mtHSP70-p53 complex, thereby relieving the inhibitory effect of mtHSP70 on p53. PRIMA-1^MET^ binds and modifies mutant p53 to restore the proper protein conformation of p53 and reactivate the wild-type function of mutant p53. Therefore, p53 can exhibit tumor-suppressive effects [165]. In summary, further development of drugs targeting HSP60 and mtHSP70 may provide novel insights into the treatment of various cancers.

#### Mitochondrial proteases and cancer

The UPR^mt^-induced mitochondrial matrix proteases ClpP and LONP1 maintain mitochondrial homeostasis by removing harmful proteins. As major mitochondrial proteases, ClpP and LONP1 play important roles in tumors [168, 169]. Multiple studies have demonstrated that ClpP and LONP1 levels are markedly increased in numerous cancers (Fig. 4G, H) [130, 168, 170–174].

ClpP interacts with multiple respiratory chain proteins and metabolic enzymes in mitochondria that are essential for metabolic regulation in cancer cells [172]. For example, the ClpPX complex containing ClpP binds and stabilizes the SDHB subunit of respiratory chain complex II, maintaining the normal functioning of OXPHOS and promoting the production of ATP. Inhibition of ClpPX leads to an imbalance in the mitochondrial ETC and oxidative stress, which ultimately reduces the proliferation and motility of cancer cells (Fig. 3) [171]. In addition, ClpP regulates the proliferation and invasion of breast cancer cells via a mechanism associated with the Src/PI3K/AKT cascade [173]. A previous study revealed that robust ClpP activity endows cancer cells with resistance to cisplatin [175]. Mechanistically, activation of ClpP increases the levels of ATP7A and ATP7B, which are involved in the elimination of cisplatin, and ClpP-mediated cisplatin efflux blocks the production of cisplatin-mtDNA/genomic DNA adducts, thereby inhibiting cancer cell death (Fig. 3) [175].

LONP1 has been found to be involved in tumor metabolic reprogramming, which is related to remodeling the components of the ETC and antagonizing cellular senescence [176]. AKT-mediated phosphorylation of LONP1 increases its protease activity. Subsequently, LONP1 ensures the stabilization of ETC complex II and complex V and, thus, protects cancer cells from damage caused by ROS accumulation [177, 178]. In fact, ClpP and LONP1 coordinately regulate mitochondrial bioenergetics in cancer, which is reflected in the observation that ClpP and LONP1 have many common substrates. The substrates regulated by ClpP and LONP1 participate in processes such as OXPHOS, the TCA cycle, and amino acid and lipid metabolism [179]. LONP1 reduces the sensitivity of colon cancer cells to apoptosis and stimulates EMT in pancreatic cancer cells (Fig. 3) [170, 174]. Furthermore, LONP1 binds and stabilizes the HSP60-mtHSP70 complex, thereby facilitating HSP60-mediated p53 inhibition and promoting cancer cell survival [180]. The application of proteasome inhibitors inhibits the progression of multiple myeloma, whereas upregulation of LONP1 decreases the efficacy of proteasome inhibitors. Mechanistically, LONP1 functions outside mitochondria to partially compensate for the lack of proteasome activity, reducing the level of damaged intracellular proteins [181]. In addition, elevated LONP1 expression increases the level of ROS to promote the production of inflammatory cytokines, including TGF-β and IL-6, thus boosting the activation of M2 macrophages and establishing an immunosuppressive tumor microenvironment (Fig. 3) [182].

Cancer cells exploit the functions of ClpP and LONP1 in mitochondrial homeostasis and energy metabolism to accelerate their own invasion and metastasis. Several studies have proven that high levels of ClpP and LONP1 in colorectal cancer, prostate cancer, breast cancer and melanoma are notably correlated with poor prognosis in cancer patients (Fig. 4G, H) [173, 176, 179]. Accordingly, targeting ClpP and LONP1 is anticipated to reveal a new therapeutic perspective for cancer due to the oncogenic functions of these proteins [183, 184]. Indeed, inhibition of LONP1 mediated by triterpenoids leads to alterations in normal mitochondrial morphology and dysregulation of mitochondrial function, which ultimately triggers the death of cancer cells [185]. Although its activation plays a crucial role in maintaining cancer cell proteostasis, the oncogenic effects of ClpP may be dose dependent. Dysregulation of ClpP also disrupts proteostasis [186]. Previous studies have shown that ONC201 and its TR compound analogs, which belong to the imipridone class of small molecules, can specifically bind to ClpP, consequently inhibiting the proliferation of cancer cells [187]. Mechanistically, imipridones noncovalently bind ClpP and cause structural changes in ClpP, which induce its hyperactivation. Subsequently, hyperactivated ClpP accelerates the degradation of ETC substrates to interfere with mitochondrial structure and function, thus killing cancer cells [186]. Additionally, dysfunction of ClpP can be caused by acyldepsipeptide analogs, leading to nonspecific hydrolysis of model substrates of ClpP, thereby triggering caspase-dependent apoptotic cell death [188].

## The roles of the UPR^mt^ in physiological processes and other diseases

Accumulating studies have indicated that the UPR^mt^ is related to many physiological and pathological processes as well as human diseases. The UPR^mt^ plays an important role in aging, the immune response, cancer, heart disease and neurodegenerative diseases [46, 189–194]. In *C. elegans*, the transcriptional program induced by UPR^mt^ involves numerous genes, which are enriched in mitochondrial chaperones, OXPHOS complex assembly factors and components, and glycolytic genes [4]. UPR^mt^ also boosts the expansion of the mitochondrial network, which is active during normal development, thus satisfying the physiological requirements of individual cells in *C. elegans* [195]. CBP-1 in nematodes and CBP/p300 in mammals are involved in the transcriptional activation of UPR^mt^ genes, thereby promoting extension of the lifespan and enhancement of immune responses [42]. The roles of the UPR^mt^ in various human diseases have been gradually revealed. For instance, in decompensated cirrhosis, aging liver cells show the characteristics of mitochondrial dysfunction and an impaired UPR^mt^. ClpP, an effector in the UPR^mt^, promotes the elimination of ROS and, thus, delays the senescence of liver cells [196]. The weak regenerative ability of hematopoietic stem cells (HSCs) is associated with inactivation of the UPR^mt^ effector protein SIRT7, and SIRT7 expression is significantly downregulated in senescent HSCs, demonstrating that UPR^mt^-mediated dysregulation of cell metabolism is one reason for the senescence of HSCs [63]. In contrast, during the transition of HSCs from quiescence to proliferation, UPR^mt^ is activated to promote metabolic adaptation [197]. Evidence indicating that the enhancement of UPR^mt^ activity inhibits the death of cardiomyocytes induced by chronic pressure overload demonstrates the cardioprotective function of the UPR^mt^ [22]. Activation of the PGC-1α/ATF5 axis is beneficial for the alleviation of pathological cardiac hypertrophy [198]. Activation of UPR^mt^ signaling promotes neurogenesis in the brains of mice with amyotrophic lateral sclerosis (ALS) [23]. Additionally, the UPR^mt^ is involved in the differentiation of myoblasts [55, 56]. In summary, the UPR^mt^ plays a critical role in a variety of physiological processes, most of which are accompanied by slight perturbations in mitochondrial homeostasis, thus leading to UPR^mt^ activation. In turn, activated UPR^mt^ maintains mitochondrial function. Specifically, moderate mitochondrial stress contributes to activation of the UPR^mt^ and stabilization of mitochondrial function [41]. However, the UPR^mt^ also acts as a promoter of the maintenance and propagation of deleterious mtDNA. The OXPHOS defect caused by the mutant mtDNA induces the UPR^mt^. Conversely, to promote mitochondrial recovery, UPR^mt^ activation leads to intracellular accumulation of harmful mtDNA, which ultimately results in cellular dysfunction. This harmful mtDNA hijacks the UPR^mt^ to facilitate its own dissemination [199, 200].

## Conclusions

As an indispensable cytoprotective mechanism, activation of the UPR^mt^ promotes the recovery of mitochondria from damage, maintains proteostasis, remodels the ETC, and eliminates accumulated ROS in response to various intracellular and extracellular stresses. Mitocytosis, a migrasome-mediated mitochondrial quality control mechanism, maintains mitochondrial membrane potential and mitochondrial respiration by removing damaged mitochondria from migrating cells [201]. It is worth exploring whether there is an association between UPR^mt^ and mitocytosis. In a sense, the UPR^mt^ in *C. elegans* is very similar to the UPR^mt^ in mammals. In both, the efficiency of mitochondrial protein transport determines the activation state. In addition, both of these UPR^mt^s involve intricate cell signaling pathways and dynamic epigenetic regulation. However, the UPR^mt^ in mammals is more complex than the UPR^mt^ in *C. elegans* because it involves more factors and regulatory branches. Although the UPR^mt^ in *C. elegans* is well understood, the UPR^mt^ in mammals needs more exploration. What are the other potential branches of the mammalian UPR^mt^? What functions do these branches have? Is there any crosstalk between the different branches of the UPR^mt^, resulting in an extensive signaling network? What is the precise nature of the UPR^mt^-induced transcriptional program, and what roles do these transcripts play? Is there a signaling node connecting the UPR^mt^, UPR^ER^ and HSR that regulates mitochondrial, ER and cytoplasmic homeostasis? The answers to these questions require further research.

The UPR^mt^ has emerged as a protective response in various human diseases, including neurodegenerative diseases [202]. Alzheimer’s disease (AD) is a multifactorial brain disorder characterized by loss of memory and aggregation of two insoluble proteins, including tau neurofibrillary tangles and β-amyloid plaques [203]. Mitochondrial dysfunction, such as changes in mitochondrial enzyme activity, damaged mitochondrial ultrastructure, excessive ROS generation, altered mtDNA, reduced mitochondrial oxygen consumption and mitophagy impairment, is a common pathological hallmark in AD patients [204]. Several lines of evidence suggest that UPR^mt^ is associated with the progression of familial and sporadic AD [192, 205]. Activation of UPR^mt^ was observed in the brains of APP/PS1 transgenic mice and SHSY5Y cells treated with Aβ [206]. Recently, an important study identified a conserved mitochondrial stress response feature in AD patients and AD animal models [207]. More importantly, pharmacological or genetic activation of UPR^mt^ attenuated cognitive impairment and decreased deposition of Aβ in an AD model [207]. Therefore, UPR^mt^ is important for the maintenance of mitochondrial proteostasis and provides potential targets for AD therapy.

Accumulating evidence indicates that the UPR^mt^ is activated in many types of tumors. The UPR^mt^ promotes the development of cancer and boosts its progression through various mechanisms. Cancer cells utilize signaling molecules and transcriptional products in the UPR^mt^, such as ATF5, HSP60 and ClpP, to promote their proliferation, growth, invasion and metastasis. Therefore, targeting components of the UPR^mt^ may be a potential, reliable, and effective method for the treatment of cancer [129, 168, 208]. For example, CP-d/n-ATF5-S1, a cell-penetrating peptide, has been exploited as an inhibitor of ATF5. CP-d/n-ATF5-S1 inhibits tumor growth by inducing apoptosis and has demonstrated excellent anticancer effects against glioblastoma, melanoma, prostate cancer and triple-negative breast cancer in a series of in vitro and in vivo experiments [208]. The relationship between UPR^mt^ and cancer needs further interpretation. Can the UPR^mt^ be activated only by mitochondrial dysregulation, or can cancer cells also promote constitutive activation of the UPR^mt^ (via, for example, upstream regulator-mediated signaling activation and epigenetic changes in UPR^mt^-related components)? Which oncogenes and tumor suppressor genes interact with the components of the UPR^mt^ in cancer? Although the role of the UPR^mt^ in cancer biology has been clarified through many years of research, more hidden mysteries are still awaiting exploration.

## Data Availability

Not applicable.

## Abbreviations

ACC: Adrenocortical carcinoma
BLCA: Bladder urothelial carcinoma
BRCA: Breast invasive carcinoma
CESC: Cervical squamous cell carcinoma and endocervical adenocarcinoma
CHOL: Cholangiocarcinoma
COAD: Colon adenocarcinoma
DLBC: Lymphoid neoplasm diffuse large B-cell lymphoma
ESCA: Esophageal carcinoma
GBM: Glioblastoma multiforme
HCC: Hepatocellular carcinoma
HNSC: Head and neck squamous cell carcinoma
KICH: Kidney chromophobe
KIRC: Kidney renal clear cell carcinoma
KIRP: Kidney renal papillary cell carcinoma
LAML: Acute myeloid leukemia
LGG: Brain lower grade glioma
LIHC: Liver hepatocellular carcinoma
LUAD: Lung adenocarcinoma
LUSC: Lung squamous cell carcinoma
MESO: Mesothelioma
OV: Ovarian serous cystadenocarcinoma
PAAD: Pancreatic adenocarcinoma
PCPG: Pheochromocytoma and paraganglioma
PRAD: Prostate adenocarcinoma
READ: Rectum adenocarcinoma
SARC: Sarcoma
SKCM: Skin cutaneous melanoma
STAD: Stomach adenocarcinoma
TGCT: Testicular germ cell tumors
THCA: Thyroid carcinoma
THYM: Thymoma
UCEC: Uterine corpus endometrial carcinoma
UCS: Uterine carcinosarcoma
UVM: Uveal melanoma
AD: Alzheimer’s disease
ALS: Amyotrophic lateral sclerosis
ATF5: Activating transcription factor 5
ATFS-1: Activating transcription factor associated with stress-1
*C. elegans*: *Caenorhabditis elegans*
ClpP: Caseinolytic mitochondrial matrix peptidase proteolytic subunit
DN-ATF5: Dominant-negative ATF5 mutant
DTHIB: Direct Targeted HSF1 InhiBitor
eIF2α: α Subunit of eukaryotic translation initiation factor 2
EMT: Epithelial-mesenchymal transition
ER: Endoplasmic reticulum
ERα: Estrogen receptor α
ETC: Electron transport chain
FUNDC1: FUN14 domain containing 1
GCN2: General control nonderepressible 2
H3K9me2: H3K9 dimethylation
HRI: Heme-regulated inhibitor
HSCs: Hematopoietic stem cells
HSF1: Heat shock factor 1
HSP60: Heat shock protein 60
HSR: Heat shock response
IGFBP7: Insulin-like growth factor-binding protein 7
ISR: Integrated stress response
LONP1: Lon peptidase 1
MAM: Mitochondria associated membranes
Met-tRNA_i_: Methionylated initiator transfer RNA
mitophagy: Mitochondrial autophagy
mtDNA: Mitochondrial DNA
mtHSP70: Mitochondrial heat shock protein 70
MTS: Mitochondrial targeting sequence
NLS: Nuclear localization sequence
OXPHOS: Oxidative phosphorylation
PERK: PKR-like endoplasmic reticulum kinase
PGAM5: Phosphoglycerate mutase 5
PKR: Protein kinase R
polyQ: Polyglutamine repeat protein
ROS: Reactive oxygen species
*S. cerevisiae*: *Saccharomyces cerevisiae*
SSBP1: Single-strand DNA binding protein 1
TCA: Tricarboxylic acid
uORFs: Upstream open reading frames
UPR^ER^: Endoplasmic reticulum unfolded protein response
UPR^mt^: Mitochondrial unfolded protein response
